# Efficacy and safety of ferric carboxymaltose versus ferrous sulfate for iron deficiency anemia during pregnancy: subgroup analysis of Korean women

**DOI:** 10.1186/s12884-018-1817-y

**Published:** 2018-08-28

**Authors:** Jae-Yoon Shim, Moon Young Kim, Young Ju Kim, Young Lee, Jeong Jae Lee, Jong Kwan Jun, Jong Chul Shin, Yong Kyoon Cho, Keun Young Lee, Ahm Kim, Tae-Bok Song

**Affiliations:** 1 Department of Obstetrics and Gynecology, Asan Medical Center, University of Ulsan College of Medicine, 88 Olympic-ro 43-gil, Songpa-gu, Seoul, 05505 Korea; 2 Department of Obstetrics and Gynecology, Cheil General Hospital and Women’s Healthcare Center, Dankook University College of Medicine, Seoul, Korea; 3 Department of Obstetrics and Gynecology, Ewha Womans University School of Medicine, Seoul, Korea; 4 Department of Obstetrics and Gynecology, Seoul St. Mary’s Hospital, College of Medicine, The Catholic University of Korea, Seoul, Korea; 5 Department of Obstetrics and Gynecology, Soonchunhyang University College of Medicine, Seoul Hospital, Seoul, Korea; 6 Department of Obstetrics and Gynecology, Seoul National University College of Medicine, Seoul, Korea; 7 Department of Obstetrics and Gynecology, Sanggyepaik Hospital, Inje University College of Medicine, Seoul, Korea; 8 Department of Obstetrics and Gynecology, Hallym University College of Medicine, Kangnam Sacred Heart Hospital, Seoul, Korea; 9 Department of Obstetrics and Gynecology, Chonnam National University Medical School, Gwangju, Korea

**Keywords:** Intravenous iron, Iron deficiency, Anemia, Pregnancy, Korea, Ferric carboxymaltose, Clinical trial

## Abstract

**Background:**

We performed a post-hoc subgroup analysis in Korean women who participated in the Phase III FER-ASAP (FERric carboxymaltose-Assessment of SAfety and efficacy in Pregnancy) study to compare the efficacy and safety of ferric carboxymaltose (FCM) with oral ferrous sulfate (FS).

**Methods:**

Pregnant Korean women (gestational weeks 16–33) with iron-deficiency anemia (IDA) were randomized 1:1 to FCM (*n* = 46; 1000–1500 mg iron) or FS (*n* = 44; 200 mg iron/day) group for 12 weeks. The primary objective was to compare the mean hemoglobin (Hb) increase at week 3; secondary objectives included change in iron parameters, quality of life (QoL), and safety.

**Results:**

Baseline characteristics of the Korean subgroup were consistent with those of non-Korean FER-ASAP population except for lower body-mass index and higher maternal age. Hb level increases were comparable between the two treatment groups in Korean women at week 3 (FCM 1.23 ± 0.89 g/dL vs FS 1.14 ± 1.72 g/dL). Iron parameters improved over time as secondary endpoints were significantly in favor of FCM. In terms of QoL, FCM treatment significantly improved the mental and physical components as well as vitality prior to delivery. Both treatments were well tolerated.

**Conclusions:**

FCM provided significantly greater improvements in iron parameters and QoL compared to FS in the Korean subgroup. FCM may be a preferable alternative to currently available treatments for IDA during pregnancy.

## Background

According to worldwide estimates, 30% of women of reproductive age are anemic and at least half of these cases are attributed to iron deficiency (ID) [1–3]. The prevalence of anemia is 14–52% in women without iron supplementation and 25% even with supplementation, depending on iron dosage [4]. Throughout pregnancy, total iron requirements increase to meet the major hematologic changes of the mother and the increasing demands of the growing fetus [5]. This increased demand for iron puts the mother and offspring at risk of developing iron-deficiency anemia (IDA). IDA during pregnancy is associated with an increased risk of preterm birth, low birthweight, fetal growth restriction, and increased newborn and maternal mortality. Furthermore, ID may predispose a person to postpartum IDA, peripartum blood transfusion, infections, and precipitate heart failure [6].

IDA during pregnancy is often treated with oral iron supplement. However, due to gastrointestinal side effects such as nausea, vomiting, and constipation, compliance is often poor and results in subsequent discontinuation [4, 7]. As such, intravenous iron administration are increasingly being recommended for women who are non-compliant with oral iron, have severe IDA, or those who require rapid intervention [6, 8]. Ferric carboxymaltose (FCM; Ferinject^®^) is a dextran-free parenteral iron preparation that allows rapid administration of weekly high single doses of iron and had been approved for the treatment of IDA by the UK Medicines and Healthcare products Regulatory Agency in 2007, US Food and Drug Administration (FDA) in 2013, and Korea FDA in 2010.

In the FER-ASAP (FERrric carboxymaltose-Assessment of SAfety and efficacy in Pregnancy) study, pregnant women (gestational weeks 16–33) with IDA were randomized in a 1:1 ratio to FCM or ferrous sulfate (FS) for 12 weeks [9]. Hemoglobin (Hb) levels improved at comparable rates in both treatments; however, significantly higher number of women achieved anemia correction within a shorter time frame with FCM (84%) than with FS (70%; *P* = 0.031). FCM treatment significantly improved the quality of life (QoL) from baseline up to delivery, and there were markedly fewer gastrointestinal treatment-related adverse events (AEs) with FCM (11%) than with FS (15%).

Geographic variations in anemia prevalence are attributed to differences in iron nutrition and socioeconomic status; however, variations in the tolerability profile of FCM between populations (according to race) have not been investigated. Approximately 40% of the women randomized and treated in the FER-ASAP study were from Korea, where the prevalence of IDA during pregnancy is reported as 15–25% [10]. Notably, total fertility rate of Korea has continued to decrease, and is now one of the lowest in the world (1.05 in 2017). With the dramatically decreasing number of pregnant women in Korea, improving the health status of pregnant women is an ever-more important issue in terms of nation-wide investment in health, and the necessity to evaluate the various anemia treatment methods.

Therefore, we performed a post –hoc subgroup analysis of the efficacy and safety of FCM in pregnant Korean women who participated in the FER-ASAP study.

## Methods

### FER-ASAP study design and participants

Details of the study design and participants of the FER-ASAP trial have been described previously [9]. Briefly, FER-ASAP was a Phase IIIb, randomized, open-label, international study (Clinicaltrials.gov, identifier; NCT01131624) conducted in Australia, Switzerland, Sweden, Turkey, Korea, Singapore, and Russia. Participants were randomized in a 1:1 ratio to receive intravenous FCM (1000–1500 mg iron) or oral FS (200 mg iron, 100 mg capsules taken twice daily). FCM dosing regimens were stratified according to pre-pregnancy bodyweight and Hb levels (Table 1) and all infusions were completed by week 3, whereas oral FS was taken daily for 12 weeks. Pregnant women aged ≥18 years (gestational weeks 16–33), and with serum ferritin levels ≤20 ng/mL and IDA (defined as Hb 8.0–10.4 g/dL for gestation weeks 16–26 or Hb ≤11.0 g/dL for gestation weeks 27–33) were eligible for this study. The study protocols were approved by the institutional review boards of the participating centers and were conducted in accordance with Good Clinical Practice and the Declaration of Helsinki. All study participants reviewed and voluntarily signed the written informed consent form prior to enrollment.

**Table 1:** Ferric carboxymaltose dose selection

Hb (g/dL)	Pre-pregnancy weight (kg)^a^	
	< 66 kg	≥66 kg
8–9	3 × 500 mg iron as FCM	1 × 1000 mg iron as FCM
		1 × 500 mg iron as FCM
9.1–10.4	2 × 500 mg FCM	1 × 1000 mg FCM

*FCM* ferric carboxymaltose, *Hb* hemoglobin

^a^If pre-pregnancy weight was not known, first trimester weight was used for dosing

### Endpoints and assessments

The primary objective of this subgroup analysis was to compare the efficacy of intravenous FCM with oral FS as treatment for IDA in pregnant Korean women during their second and third trimesters. The primary efficacy endpoint was the change in Hb from baseline to week 3. Secondary efficacy endpoints included change in Hb and other serum iron parameters as well as the proportion of women who achieved anemia correction (defined as Hb ≥11.0 g/dL). Secondary objectives were determining the effect of FCM on QoL and the safety and tolerability of FCM in pregnant women and their newborns. Further details on assessments can be found in the previously published FER-ASAP study [9].

### Statistics

Statistical analyses followed the same protocol as described previously [9].

A sample size of 120 women per treatment arm was considered as sufficiently powered to detect cross-group differences in Hb levels at week 3 versus baseline (α = 0.05, two-sided; 90% power). The sample size was increased to 125 women per treatment arm to account for the expected discontinuation at week 3, thereby bringing the total planned study population size to 250. Efficacy analyses were conducted on the full analysis set comprising all randomized women who received at least one dose of study treatment and had at least one efficacy assessment available at baseline and within the study period. The safety set included all women who received at least one dose of study treatment. For all analyses, missing data were treated as missing, and no imputation was performed. Post-hoc statistical analyses of the study groups at baseline, FCM vs. FS in Korean subgroup, FCM vs. FS in non-Korean subgroup, Korean vs. non-Korean in FCM, and Korean vs. non-Korean in FS, was performed using Student t-test, chi-square test, or Fisher’s exact test as appropriate. Fisher’s exact test was used to compare the incidence of adverse events between study groups, and data from newborns were analyzed using Student’s t-test.

## Results

### Patient disposition and baseline characteristics

**Fig. 1:**
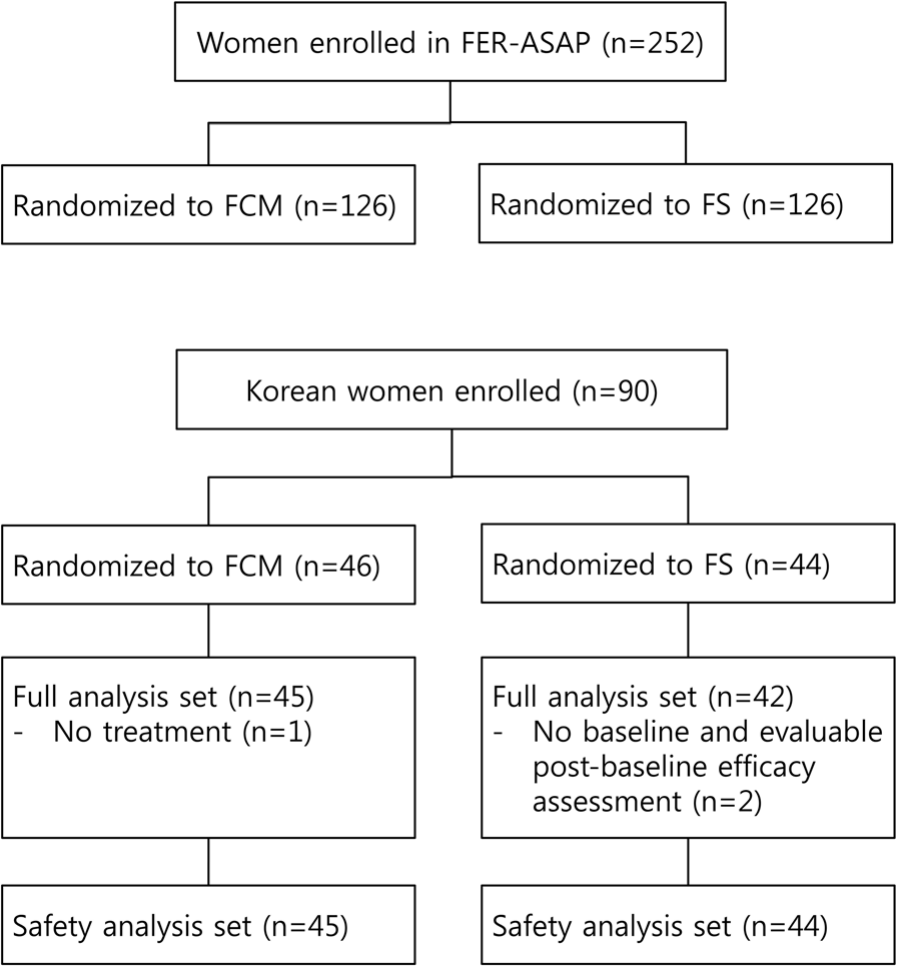
Disposition of participants. FCM, ferric carboxymaltose, FS, ferrous sulfate

Of the 252 pregnant women enrolled and randomized in the primary FER-ASAP study, 90 were Korean and were treated with FCM (*n* = 46) or FS (*n* = 44) across nine centers in Korea from 2010 to 2014 (Fig. 1). Because three women did not fulfill the efficacy analysis set (Fig. 1), 87 pregnant women were included in the full analysis set (FCM, *n* = 45; FS, *n* = 42) and 89 in the safety analysis set (FCM, *n* = 45; FS, *n* = 44). One woman randomized to FCM was excluded in the safety analysis set because she did not receive any study treatment. The baseline demographics of the two treatment groups were well-matched (Table 2). However, compared with non-Korean women, Korean women had lower body-mass index and higher maternal age (Table 2).

**Table 2:** Baseline demographics

	Korean subgroup		Non-Korean subgroup	
	FCM (*n* = 45)	FS (*n* = 44)	FCM (*n* = 78)	FS (*n* = 80)
Age, years	34.5 ± 4.8	33.4 ± 4.9	29.6 ± 5.6	29.5 ± 5.2
Pre-pregnancy weight, kg	53.9 ± 8.0	54.7 ± 8.7	62.4 ± 12.2	58.9 ± 11.4
Pre-pregnancy BMI, kg/m^2^	23.7 ± 2.7	25.0 ± 3.3	25.6 ± 4.3	24.6 ± 3.9
Baseline weight, kg	61.3 ± 7.7	63.8 ± 9.5	70.1 ± 13.5	66.5 ± 12.0
Baseline weight category, n (%)				
< 66 kg	35 (77.8)	28 (63.6)	34 (43.6)*	40 (50.0)
≥ 66 kg	10 (22.2)	16 (36.4)	44 (56.4)	40 (50.0)
Overall total dose, mg	1066 ± 172	12,286 ± 3596	1006 ± 170	11,777 ± 5124
Baseline Hb level, g/dL	9.68 ± 0.92	9.84 ± 1.55	9.82 ± 0.79	9.98 ± 0.89
Baseline Hb level category, n (%)				
≤ 9.0 g/dL	11 (24.4)	6 (13.6)	11 (14.1)	11 (13.8)
> 9.0 g/dL	34 (75.6)	38 (86.4)	67 (85.9)	68 (85.0)
Baseline serum ferritin				
≤ 10 ng/mL	36 (80.0)	28 (63.6)	62 (79.5)	58 (72.5)
> 10 ng/mL	9 (20.0)	15 (34.1)	14 (17.9)	22 (27.5)
Gestational age category				
16 to < 20 weeks	3 (6.7)	2 (4.5)	9 (11.5)	7 (8.8)
20 to < 33 weeks	40 (88.9)	40 (90.9)	65 (83.3)	70 (87.5)
≥ 33 weeks	2 (4.4)	2 (4.5)	4 (5.1)	3 (3.8)

Results presented here are mean (and standard deviation) unless otherwise stated

*BMI* body mass index, *FCM* Ferric carboxymaltose, *FS* ferrous sulfate

*P < 0.05 vs. Korean subgroup FCM. Values with no asterisk have no statistically significant difference when compared with other three values in the same row using Student’s t-test, chi-square test, or Fisher’s exact test

### Efficacy analysis

**Fig. 2:**
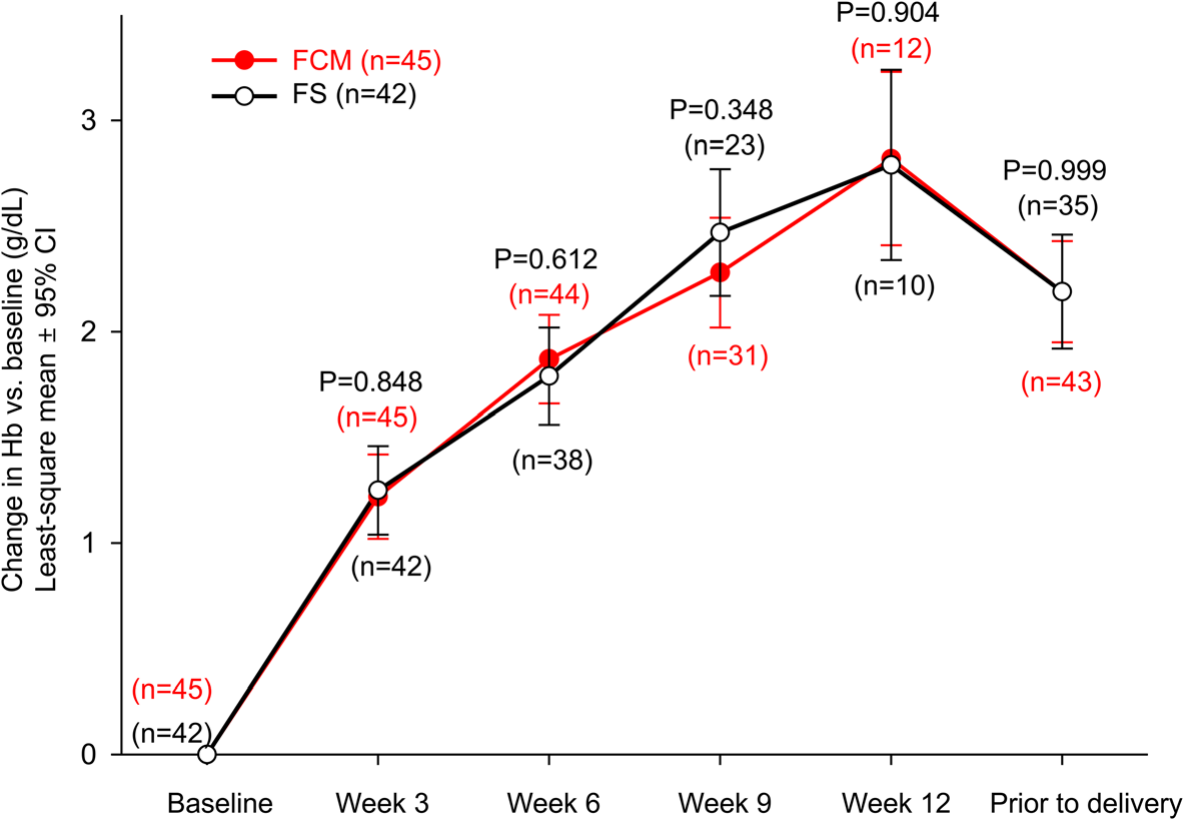
Changes in hemoglobin levels from baseline over time. CI, confidence interval; FCM, ferric carboxymaltose, FS, ferrous sulfate; Hb, hemoglobin

In pregnant Korean women, Hb level was increased at each visit (vs. baseline) throughout the study period in both treatment groups (Fig. 2). FCM led to a greater improvement in Hb status from baseline to week 3 than FS; however, the difference was not statistically significant and the primary endpoint was not met (least-squares mean difference: − 0.03, 95% confidence interval [CI]: − 0.32, 0.26; *P* = 0.848). Nevertheless, except at week 9, mean changes in Hb level over time in the FCM group were consistently higher compared with that in the FS group. Furthermore, for the Korean subgroup, women treated with FCM were more likely to achieve anemia correction (Hb ≥ 11.0 g/dL) than those treated with FS (93.3% vs 85.7%; *P* = 0.304), albeit not statistically significant.

**Fig. 3:**
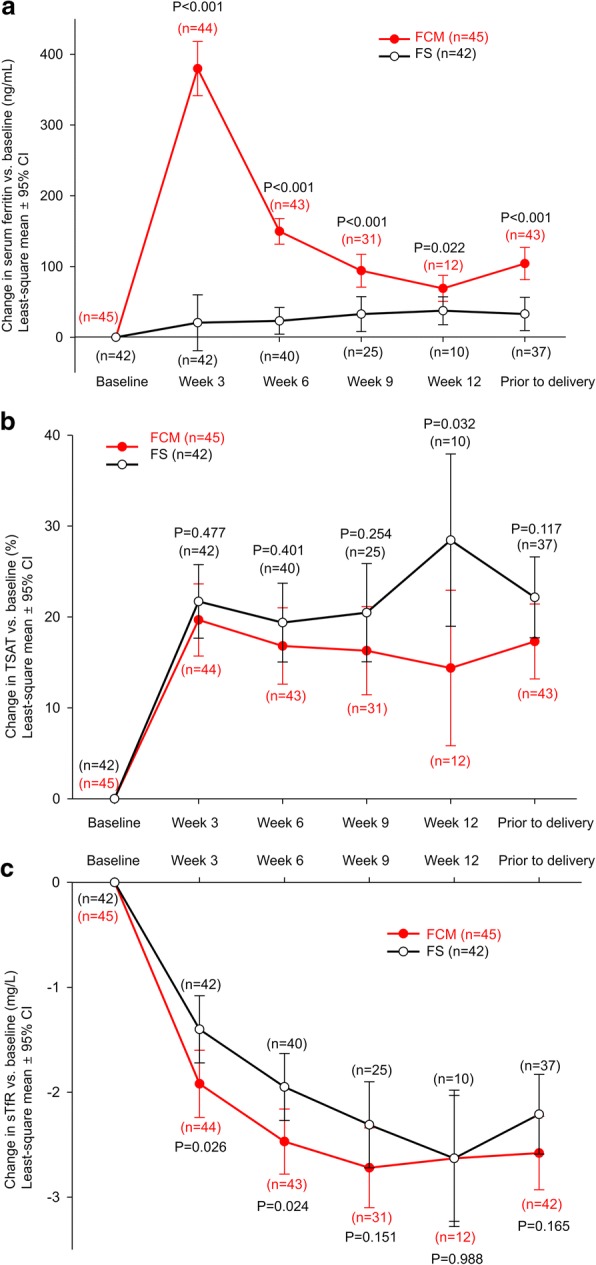
Changes from baseline over time in **a** serum ferritin; **b** transferrin saturation; **c** soluble transferrin receptor. CI, confidence interval; FCM, ferric carboxymaltose, FS, ferrous sulfate; sTfR, soluble transferrin receptor; TSAT, transferrin saturation

Comparative analysis of secondary hematological parameters demonstrated significantly higher increases in serum ferritin at each visit (vs. baseline) for FCM versus FS treatment throughout the study period (Fig. 3a). Transferrin saturation (TSAT) levels were similar in both treatment groups from baseline up to week 9; however, at week 12, the mean TSAT levels for the FCM group were significantly greater in comparison with that of the FS group (least-squares mean difference − 14.07; 95% CI –26.85, − 1.28; *P* = 0.032; Fig. 3b). Soluble transferrin receptor levels decreased in both treatment groups, with significantly more rapid and greater decreases in FCM treatment at week 3 (least-squares mean difference − 0.52; 95% CI –0.98, − 0.07; *P* = 0.026) and week 6 (least-squares mean difference − 0.52; 95% CI –0.98, − 0.07; *P* = 0.024; Fig. 3c).

### Quality of life

**Fig. 4:**
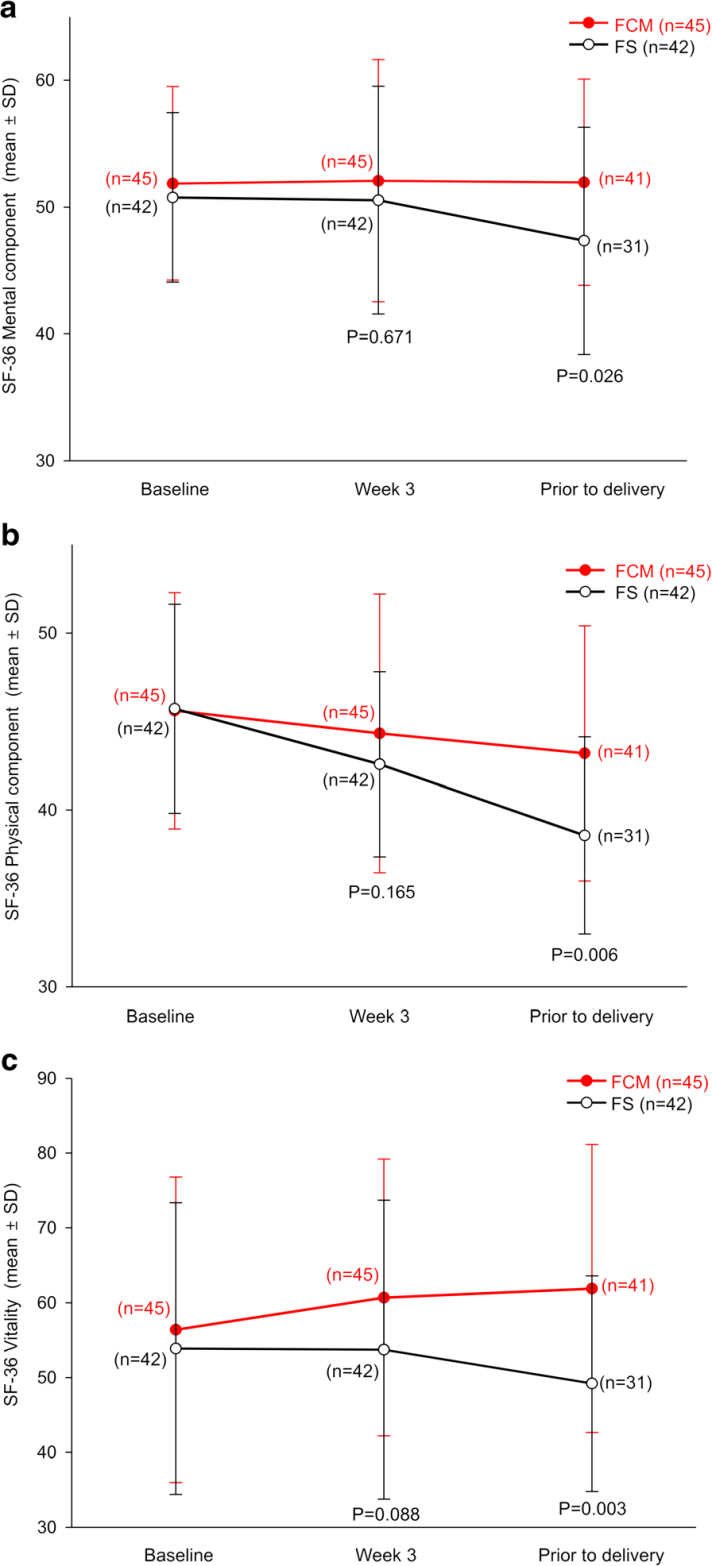
Changes in SF-36 component scores over time for **a** mental **b** physical, and **c** vitality components. *P* values have been calculated from the mean difference between baseline and assessment points. FCM, ferric carboxymaltose, FS, ferrous sulfate; SF-36, 36-item short-form health survey

According to SF-36 health scores, pregnant Korean women treated with FCM achieved significant and clinically relevant improvements over FS in all three 36-item short-form questionnaires (mental, physical, and vitality) prior to delivery (mean score change, mental, FCM 0.09 vs. FS -3.41, *P* = 0.026; physical, FCM − 2.41 vs. FS -7.16, *P* = 0.006; vitality, FCM 2.41 vs. FS -7.16, *P* = 0.003) (Fig. 4a, b, c).

### Safety evaluation

Within the Korean subgroup, the mean total doses administered for FCM and FS were 1067 ± 172 mg and 12,286 ± 3596 mg, respectively, and the mean durations of exposure were the two groups were 6.5 ± 4.1 and 66.1 ± 20.4 days, respectively. The total incidences of treatment-emergent adverse events (TEAEs) were similar in both groups (FCM 83; FS 70; total 153) and were reported in 27 women (60.0%) in the FCM group and 28 women (63.6%) in the FS group (Table 3). The majority of events were mild, and there were no severe AEs.

**Table 3:** Treatment-emergent adverse events occurring in Korean and non-Korean subgroup

Adverse event, n (%)	Korean subgroup		Non-Korean subgroup	
	FCM (*n* = 45)	FS (*n* = 44)	FCM (*n* = 78)	FS (*n* = 80)
Any adverse event	27 (60.0)	28 (63.6)	33 (42.3)	22 (27.5)
Severe	0 (0)	0 (0)	0 (0)	2 (2.5)
Leading to discontinuation	0 (0)	2 (4.5)	1 (1.3)	5 (6.3)
Outcome of death	0 (0)	0 (0)	0 (0)	0 (0)
Any treatment-related adverse event	4 (8.9)	8 (18.2)	10 (12.8)	11 (13.8)
Severe	0 (0)	0 (0)	0 (0)	1 (1.3)
Leading to discontinuation	0 (0)	2 (4.5)	1 (1.3)	4 (5.0)

Values with no asterisk have no statistically significant difference when compared with other three values in the same row using Fisher’s exact test

*FCM* ferric carboxymaltose, *FS* ferrous sulfate

Overall, common TEAEs in the Korean subgroup included headache (7.9%), dyspepsia (7.9%), and constipation (6.7%); the most common TEAEs according to system organ class were “pregnancy, puerperium and perinatal conditions” in the FCM group (20 events in 16 women [35.6%]) and “gastrointestinal disorders” in the FS group (27 events in 14 women [31.8%]). The most common treatment-related adverse events (TRAEs) were headache and dizziness in FCM (experienced by three women [6.7%]) and nausea and diarrhea in FS (in four women [(9.1%]). There were markedly higher rates of gastrointestinal TRAEs reported with FS (19 events) compared with FCM treatment (one event; Table 4), and two women discontinued treatment with FS because of gastrointestinal TRAEs. No hypophosphatemia TEAEs were reported during this study in pregnant Korean women.

**Table 4:** Treatment-related adverse events (experienced by ≥1 pregnant Korean women) by MedDRA primary system organ class and preferred term

Any treatment-related adverse event, n (%)	FCM (*N* = 45)	FS (*N* = 44)	*P* value
Total number of patients	4 (9)	8 (18)	0.23
Total adverse event	9	19	
Gastrointestinal disorders			
Nausea	1 (2)	4 (9)	0.20
Vomiting	0 (0)	2 (5)	0.24
Constipation	0 (0)	3 (7)	0.12
Diarrhea	0 (0)	4 (9)	0.06
Abdominal pain upper	0 (0)	3 (7)	0.12
Dyspepsia	0 (0)	3 (7)	0.12
Nervous system disorders			
Headache	3 (7)	0 (0)	0.24
Dizziness	3 (7)	0 (0)	0.24
General disorders			
Fatigue	1 (2)	0 (0)	0.95
Pyrexia	1 (2)	0 (0)	0.95

According to the physician’s assessment, a single patient could appear in multiple classes. Percentages were calculated according to treatment group

Fisher’s exact test was used to compare the incidence of each adverse event between study groups

*FCM* ferric carboxymaltose, *FS* ferrous sulfate, *MedDRA* Medical Dictionary for Regulatory Activities (version 16.1)

### Newborn status

Apgar scores of the Korean newborns were similar between the two treatment groups (Table 5). Other physical parameters of newborns were also similar between the two groups, and hematological parameters were within normal range, regardless of the mother’s conditions.

**Table 5:** Newborn characteristics

	Korean subgroup (mean ± SD)		Non-Korean subgroup (mean ± SD)	
	FCM (*N* = 45)	FS (*N* = 44)	FCM (*N* = 78)	FS (*N* = 80)
Birthweight, kg	3.2 ± 0.4	3.2 ± 0.4	3.5 ± 0.5	3.5 ± 0.5
Birth length, cm	49.6 ± 2.4	49.4 ± 2.5	51.6 ± 2.5	51.5 ± 2.9
Cord hemoglobin, g/dL	14.9 ± 1.2	15.0 ± 1.3	14.7 ± 2.8	14.0 ± 2.0
Cord hematocrit, ratio	0.5 ± 0.1	0.5 ± 0.1	0.5 ± 0.1	0.5 ± 0.1
Cord serum ferritin, μg/L	269 ± 139	227 ± 117	242 ± 225	248 ± 155
1-min Apgar	9.5 ± 0.8	9.0 ± 1.4	8.2 ± 0.7	8.1 ± 1.3
5-min Apgar	9.4 ± 0.6	9.1 ± 0.8	9.0 ± 0.6	8.9 ± 0.8

Values with no asterisk have no statistically significant difference when compared with other three values in the same row using Student’s t-test

*FCM* ferric carboxymaltose, *FS* ferrous sulfate

## Discussion

Results from this FER-ASAP subgroup analysis of pregnant Korean women with IDA during late stage pregnancy showed that Hb increases were greater after FCM treatment compared with FS treatment, although the difference was not statistically significant at week 3. Secondary iron parameter measurements including serum ferritin, TSAT, and serum transferrin receptor levels were significantly better in FCM, and more women were likely to achieve anemia correction with FCM as well. In terms of QoL, FCM treatment led to significant and clinically relevant improvements compared to FS in all three SF-36 components prior to delivery.

The FER-ASAP study was the largest prospective randomized study conducted in pregnant women with IDA, and its results showed that intravenous FCM was effective and well tolerated during late-stage pregnancy [9]. In our subgroup analysis, efficacy outcomes of FCM in Korean women were generally consistent with those observed in the overall study population. However, a few major differences were observed. For example, in the overall FER-ASAP population, significantly more women achieved anemia correction within a shorter timeframe with FCM treatment (*P* = 0.031); such difference in timeframe was not statistically significant in pregnant Korean subgroup (*P* = 0.304), which is probably due to the lack of sufficient statistical power. Both oral and intravenous iron were more efficacious for correcting IDA in pregnant Korean women (89.7%) than in non-pregnant Korean women (69.6%; *P* < 0.001), despite the mean treatment duration being similar (FCM, Korean 78.4 days vs non-Korean 79.8 days; FS, Korean 71.2 days vs non-Korean 74.7 days). One possible explanation for this phenomenon is that the pregnant Korean women enrolled in this study tended to have lower pre-pregnancy and baseline weight (mean 54 kg and 60 kg, respectively) than those of the non-Korean population (mean 60 kg and 68 kg, respectively), which may also explain the higher response rate in pregnant Korean women with similar treatment duration. In this study, FCM was administered at a conservative dose, with each single dose not exceeding 15 mg iron per kg of body weight, which is lower than the current recommended dose (20 mg iron per kg of body weight). The use of the lower FCM dose in our study may have impacted the outcomes and it should be considered that use of a higher dose in accordance with the current label may lead to greater efficacy [9].

In addition to improvements in hematological parameters, FCM provided a number of QoL benefits compared with FS. Mental, physical, and vitality components of the SF-36 questionnaire showed clinically significant improvements prior to delivery in the FCM group, which is expected to provide benefits in physical stress management during pregnancy and childbirth [9]. More QoL benefits were seen in the Korean subgroup compared with the overall FER-ASAP population—the overall FER-ASAP population only showed significant improvements in vitality components (*P* = 0.025), and not in mental and physical components. These improvements in QoL parameters are generally consistent with previous studies suggesting FCM use in patients with anemia [11, 12] and may be due to the dramatically reduced burden of FCM treatment (i.e., a 12-fold lower total dose and a 10-fold shorter duration of exposure compared with FS). This is important as a higher treatment burden could lead to a decrease in compliance and adherence and lead to subsequent worsening of disease and increased healthcare costs [13].

FCM was well tolerated in Korean women during late-stage pregnancy and the incidents of TEAEs for FCM and FS treatment were generally consistent with the safety profiles observed in the primary FER-ASAP trial [9]. However, compared with non-Korean population, the incidence rates of TEAEs were significantly higher in the Korean subgroup (61.8% in Korean group vs. 34.8% in non-Korean group, *P* < 0.001) although TRAEs were similar between Korean and non-Korean groups and the frequency of AEs leading to permanent discontinuation was lower in the Korean subgroup (2.2%) than in the non-Korean subgroup (3.8%). Higher incidence of TEAEs and not TRAEs could be due to the older mean age of Korean subjects. In line with previous reports, treatment with FCM led to fewer gastrointestinal AEs than with FS [11, 14, 15]: 8 out of 44 (18.2%) pregnant Korean women using FS experienced gastrointestinal disorders, whereas TRAEs of this clinical category occurred in only 2.2% of the FCM group (*P* = 0.01). Furthermore, gastrointestinal side effects caused permanent discontinuation in two Korean women treated with oral FS. This considerable decrease in stress related to gastrointestinal disorders may be valuable for increasing the overall QoL in the latter stages of pregnancy [13].

ID and IDA during pregnancy can be further aggravated by blood loss during delivery and subsequently lead to increased risk of peripartum blood transfusion and chronic IDA [6]. In Korea, the primary mode of treatment for IDA during pregnancy has been oral iron administration and red blood cell (RBC) transfusions. Oral iron is commonly used because it is inexpensive and moderately effective. However, as many previously published studies and our Korean subgroup analysis revealed, oral iron intake was associated with a considerable amount of gastrointestinal side effects [11, 14].

Blood transfusion is a substantial alternative; however, blood transfusion is also associated with substantial risks such as transfusion-related acute lung injury, microbial contamination, possibility of incorrect transfusions, acute hemolytic allergic transfusion, and febrile non-hemolytic transfusion reactions [16]. There are special considerations for blood transfusion in Korea: first, transfusion guidelines and patient blood management programs are not commonly implemented in Korea. Second, there are growing concerns in Korea over the potential decrease in blood donations and possible blood shortages due to reduced fertility rates and increased aging population. Lastly, under the current Korean healthcare system, blood product transfusion therapy is provided at a low cost—considering per capita gross domestic product and purchasing power parity, the RBC prices in Korea were estimated to be 30–40% of those of other countries [17]. Therefore, the optimal costing model must be ensured to decrease blood component transfusion and to find the alternative treatment modality with higher cost-effectiveness.

Intravenous iron supplementation may be a preferable alternative. To date, thousands of patients have been treated with FCM in various clinical trials and recovery of iron stores has been consistently reported [11, 14, 18–20]. In patients with more severe or prolonged IDA, anemia correction using FCM consistently resulted in clinically significant increases of Hb values. In addition to the correction of hematological parameters, intravenous FCM therapy led to significant improvements of QoL and functional status. Previous studies have also suggested that FCM is associated with cost savings in various chronic IDA conditions [21–23]. For example, iron repletion with FCM for IDA in chronic heart failure patients was shown to be cost-effective when compared with placebo in Korea [24]. Further evaluation regarding the cost-effectiveness of FCM during pregnancy may be required.

Interpretation of this study is subject to some limitations. First, this was an inherent open-label study, which may introduce bias especially in terms of self-reporting QoL parameters and AEs. However, previous randomized trials have also shown that FCM showed benefits such as decreasing AEs and improving QoL [9, 11, 20]. Second, this was not a pre-specified post-hoc subgroup analysis of the FER-ASAP trial and thus could be prone to selection bias. Third, the fact that this was a Korean subgroup analysis involving 40% of the primary FER-ASAP study population would also indicate insufficient power to draw definitive conclusions. Nonetheless, the demographics and characteristics of the Korean population were generally consistent with those of the overall FER-ASAP population and the outcomes observed in the Korean population were similar to those of the overall study population. Importantly, as 40% of the total FER-ASAP populations included were Korean women and considering the special circumstances in Korea, clinical experience will be essential for providing the safety profile between intravenous and oral iron supplement in pregnant Korean women.

## Conclusions

FCM offered an efficacious and time-efficient correction of IDA in Korean women during late stages of pregnancy. Furthermore, clinically significant improvements in QoL and significantly decreased gastrointestinal side effects were observed in the Korean subgroup. In patients who are unable to tolerate oral iron or only have very short time period prior to delivery (and identification or non-correction of IDA), FCM could be an alternative solution for anemia correction with a similar safety profile to oral iron.

## Abbreviations

AEs: Adverse events
CI: Confidence interval
FCM: Ferric carboxymaltose
FDA: Food and Drug Administration
FER-ASAP: FERric carboxymaltose-Assessment of SAfety and efficacy in Pregnancy
FS: Ferrous sulfate
Hb: Hemoglobin
ID: Iron deficiency
IDA: Iron-deficiency anemia
QoL: Quality of life
RBC: Red blood cell
TEAEs: Treatment-emergent adverse events
TRAEs: Treatment-related adverse events
TSAT: Transferrin saturation
